# Lowering of lysophosphatidylcholines in ovariectomized rats by *Curcuma comosa*

**DOI:** 10.1371/journal.pone.0268179

**Published:** 2022-05-19

**Authors:** Jetjamnong Sueajai, Nareerat Sutjarit, Nittaya Boonmuen, Saranya Auparakkitanon, Nantida Noumjad, Apichart Suksamrarn, Nawaporn Vinayavekhin, Pawinee Piyachaturawat

**Affiliations:** 1 Toxicology Graduate Program, Faculty of Science, Mahidol University, Bangkok, Thailand; 2 Department of Pathology, Faculty of Medicine Ramathibodi Hospital, Mahidol University, Bangkok, Thailand; 3 Graduate Program in Nutrition, Faculty of Medicine, Ramathibodi Hospital, Mahidol University, Bangkok, Thailand; 4 Department of Physiology, Faculty of Science, Mahidol University, Bangkok, Thailand; 5 Department of Chemistry, Faculty of Science, Ramkhamhaeng University, Bangkok, Thailand; 6 Center of Excellence in Natural Products Chemistry, Department of Chemistry, Faculty of Science, Chulalongkorn University, Bangkok, Thailand; 7 Center of Excellence in Biocatalyst and Sustainable Biotechnology, Faculty of Science, Chulalongkorn University, Bangkok, Thailand; Universidade do Estado do Rio de Janeiro, BRAZIL

## Abstract

Decline of ovarian function in menopausal women increases metabolic disease risk. *Curcuma comosa* extract and its major compound, (3*R*)-1,7-diphenyl-(4*E*,6*E*)-4,6-heptadien-3-ol (DPHD), improved estrogen-deficient ovariectomized (OVX) rat metabolic disturbances. However, information on their effects on metabolites is limited. Here, we investigated the impacts of *C*. *comosa* ethanol extract and DPHD on 12-week-old OVX rat metabolic disturbances, emphasizing the less hydrophobic metabolites. Metabolomics analysis of OVX rat serum showed a marked increase compared to sham-operated rat (SHAM) in levels of lysophosphatidylcholines (lysoPCs), particularly lysoPC (18:0) and lysoPC (16:0), and of arachidonic acid (AA), metabolites associated with inflammation. OVX rat elevated lysoPCs and AA levels reverted to SHAM levels following treatments with *C*. *comosa* ethanol extract and DPHD. Overall, our studies demonstrate the effect of *C*. *comosa* extract in ameliorating the metabolic disturbances caused by ovariectomy, and the elevated levels of bioactive lipid metabolites, lysoPCs and AA, may serve as potential biomarkers of menopausal metabolic disturbances.

## Introduction

Decline of ovarian estrogen production in post-menopausal women leads to disturbances, which increase the risk of metabolic disorders, such as insulin resistance, type 2 diabetes, and cardiovascular disease [1]. These metabolic dysfunctions gradually develop with time. Currently, several omics approaches, such as metabolomics analysis of blood and tissue samples, have been applied to gain new insights into the pathophysiology of metabolic diseases and to evaluate the severity of metabolic disorders and the effectiveness of treatment [2–4].

Metabolomics is an emerging tool for investigating small molecules and biochemical metabolites. Profiles of metabolites provide essential information regarding an individual’s health status. Currently, metabolomics has been applied to gain new insight into pathophysiology of metabolic diseases. Using gas chromatography/ time-of-flight mass spectrometry (GC/TOF MS), Ma *et al* [5, 6] reported alterations of blood metabolites involved in the metabolism of several nutrients in ovariectomy (OVX)-induced obesity and bone loss, namely, increases in cholesterol, phospholipid metabolites, branched-chain amino acids, homocysteine, hydroxyproline, and 3-hydroxybutyric acid in OVX rat serum compared to those of sham-operated control. Phytoestrogen genistein was reported to be effective in restoring levels of arachidonic acid (AA), cholecalciferol, eicosapentaenoic acid, and ergocalciferol in OVX rat to control levels [7, 8]. Other pieces of evidence support the therapeutic use of phytoestrogens to improve metabolic profiles in post-menopausal women and elderly individuals [9, 10].

*Curcuma comosa* Roxb. (*C*. *comosa*) is a medicinal plant in the Zingiberaceae family. Its rhizome extract exhibits estrogenic-like activity by inducing vaginal cell cornification and increasing uterine weight [11]. Ethanol extract contains diarylheptanoids [12], among which non-phenolic (3*R*)-1,7-diphenyl-(4*E*,6*E*)-4,6-heptadien-3-ol (DPHD) is the most abundant constituent and possesses the most potent estrogenic-like activity [13, 14]. DPHD acts at the transcriptional level similar to that of estrogen [13].

Both *C*. *comosa* rhizome ethanol extract and DPHD have been reported to have other pharmacological activities, such as stimulating osteoblast cells differentiation [15] and sparing bone loss in ovariectomized (OVX) rat [16], and improve lipid status, glucose metabolism and insulin sensitivity in OVX dyslipidemia rat [17]. Recently, DPHD has been shown to exert a lipid-lowering effect by reducing visceral fat mass and adipocyte size, inhibiting lipogenesis and promoting fatty acid oxidation in OVX rat [18]. In addition, DPHD inhibits adipocyte differentiation of human bone marrow-derived mesenchymal stem cells [19], which may promote differentiation of these cells to become osteoblasts, suggesting a beneficial effect of DPHD in ameliorating the decline of osteoblast pool during aging. Both *C*. *comosa* rhizome extract and DPHD also possess antioxidant and anti-inflammation activities, which prevent impairment of vascular relaxation in estrogen-deficient animals via an ER-eNOS pathway [20]. Anti-inflammatory activity of *C*. *comosa* extract was also demonstrated in hypercholesterolemic rabbit manifesting atherosclerotic plague formation and platelet aggregation [21]. Treatment with *C*. *comosa* extract decreases expressions of proinflammatory cytokines IL-1, MCP-1, and TNF-α [22]. *C*. *comosa* extract also contains phenolic diarylheptanoids, such as (3*S*)-1-(3,4-dihydroxyphenyl)-3-hydroxy-7-phenyl-(6*E*)-6-heptene, which exhibits antioxidant activity comparable to that of vitamin C and Trolox, providing protection against cisplatin-induced nephrotoxicity [23].

As *C*. *comosa* displays a variety of pharmacological activities, in order to advance our understanding on the implications of its use as a health promoting food supplement for post-menopausal women, untargeted metabolomics have been employed in the analysis of serum lipid profiles of OVX rat compared to sham-operated control, demonstrating elevations of sphingosine-containing phospholipids, such as ceramides, ceramide-1-phosphate, and sphingomyelins in OVX rat, and that treatment with diarylheptanoid DPHD, *C*. *comosa* ethanol extract or powder, return levels of all upregulated lipids to their sham-operated control condition [24]. Although the previous liquid chromatography (LC)–MS technique could detect the majority of known lipid classes in a biological matrix [24], the diversity of metabolites in a biological sample renders it impossible to analyze simultaneously all possible metabolites using a single technique. Additionally, more abundant metabolites could obscure detection of those with lower abundance but biologically relevant.

Here, we employed untargeted metabolomics employing different extraction and analytical conditions from those previously reported [24] to investigate serum metabolic profiles of small and less hydrophobic metabolites in OVX, and in *C*. *comosa* ethanol extract- and DPHD-treated OVX rats. The findings should extend our knowledge regarding metabolic disturbances in OVX rat and the return to normal status following *C*. *comosa* extract treatment, which could be applicable to ameliorate metabolic changes associated with post-menopausal women.

## Materials and methods

### Chemicals and plant materials

Acetonitrile, water, and methanol (HPLC grade) were from Merck (Darmstadt, Germany), and amino acids from Sigma Aldrich (St. Louis, MO, USA). Formic acid (LC–MS grade) was from Fisher Scientific (Geel, Belgium) and *N*-methyl-*N*-(trimethylsilyl) trifluoroacetamide (MSTFA) from Thermo Scientific (Rockford, IL, USA).

*C*. *comosa* extract and its diarylheptanoid, (3*R*)-1,7-diphenyl-(4*E*,6*E*)-4,6-heptadien-3-ol (DPHD), were prepared as previously described [12]. Briefly, dried and ground *C*. *comosa* rhizomes were extracted three times with three volumes of hot 95% ethanol, pooled and dried *in vacuo*. The resulting dark brown viscous oil was analyzed using high-performance liquid chromatography (HPLC)-ultraviolet (302 nm) spectroscopy to determine its composition, which contained 87.5 mg of DPHD/g *C*. *comosa* extract as previously reported [24].

### Animals, treatments, and blood sample collection

**Fig 1:**
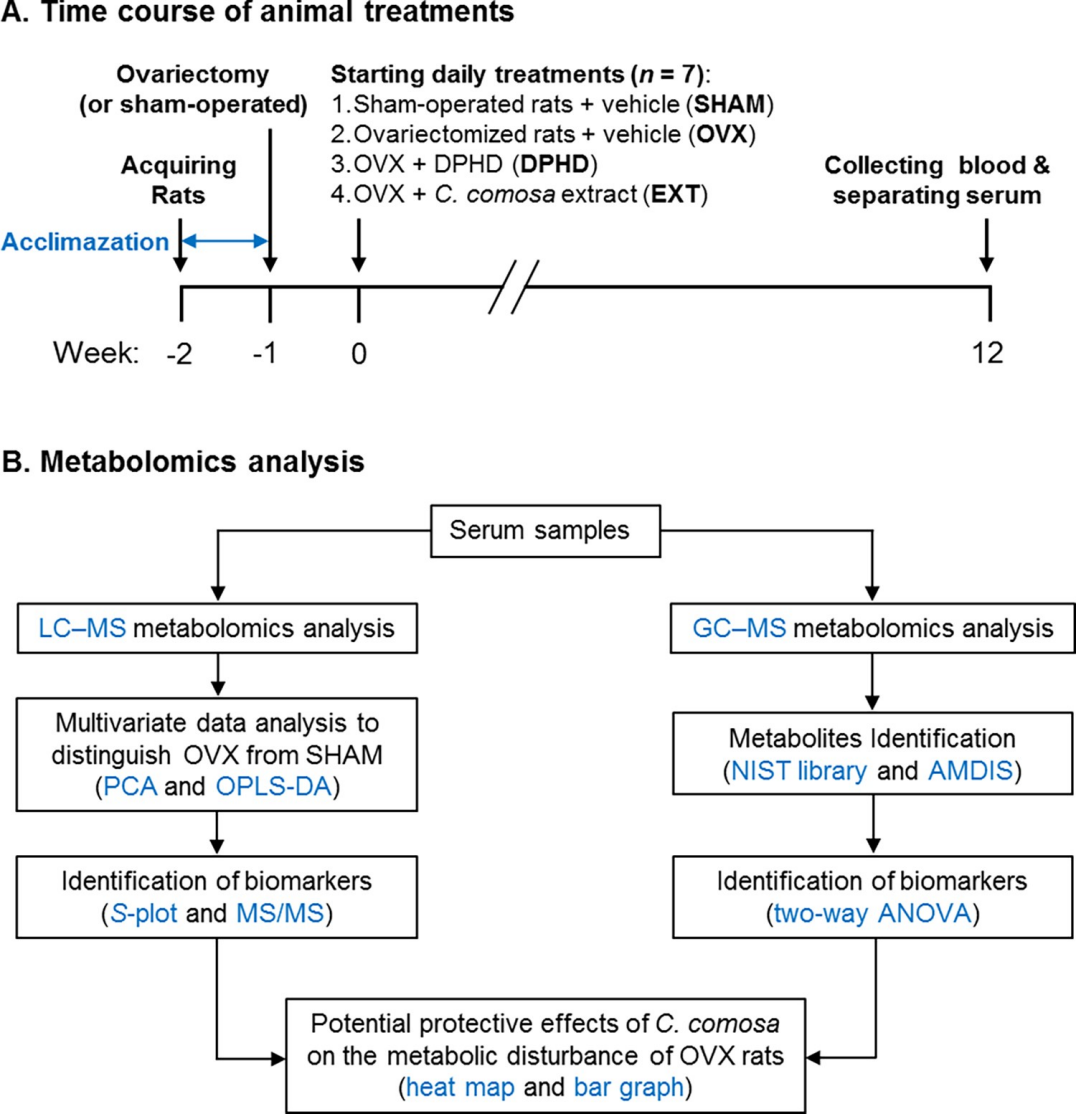
Schematic diagram of (A) experimental procedures and (B) metabolomics analysis.

Eight-week-old female Sprague-Dawley rats weighing 208 ± 10 g supplied by the National Laboratory Animal Center of Thailand (Salaya, Nakhon Pathom, Thailand) were housed in stainless steel cages under 12 h of light/dark cycle at 25°C ± 2°C with free access to rat chow pellets (Pokphand Animal Feed Co. Ltd., Bangkok, Thailand) and water *ad libitum*. After an acclimatization period of 7 days, rats were subjected to surgical bilateral ovariectomy and treatments described previously [15]. The effect of ovariectomy was confirmed from the cessation of estrous cycle as determined by a vaginal smear for 10 days (2 cycles) post-operation. The animals (7 per group) were assigned to 4 groups: (i) sham-operated control (SHAM), (ii) bilateral ovariectomy (OVX), (iii) OVX treated subcutaneously with DPHD (50 mg/kg body weight (BW)) (DPHD) or (iv) OVX treated intragastrically with *C*. *comosa* ethanol extract (500 mg/kg BW) (EXT). DPHD was dissolved in olive oil for subcutaneous injection in a final volume of 0.1–0.2 mL/rat. *C*. *comosa* extract was suspended in 1% carboxymethyl cellulose for intragastric administration at a volume of 1 mL/rat. As controls, SHAM and OVX groups were administered intragastrically with a vehicle of *C*. *comosa* extract (1% carboxymethyl cellulose). Animals received treatment once a day for 12 weeks. Body weights of rats were recorded weekly during the course of treatment. At the end of the treatment period, rats were fasted overnight and anesthetized intraperitoneally with Zoletil (20 mg/kg BW) following pre-treatment with Xylavet (5 mg/kg BW). A blood sample was collected, and serum stored at −80°C until analysis. Uterus was removed and weighed. The treatment doses and duration used in this study (Fig 1A) were based on earlier observations that these doses can protect against complications observed in OVX animals, including insulin resistance, hyperlipidemia, impairment of vascular relaxation, and bone loss [15–20]. In our study, the amount of DPHD in *C*. *comosa* extract at a dose of 500 mg/kg BW was close to 50 mg/kg BW.

The experimental protocol was approved by the Animal Care and Use Committee, Faculty of Science, Mahidol University (Protocol no. MUSC56-031-293).

### Sample preparation for metabolite analysis

Serum samples were prepared as previously described [25]. Briefly, 150 *μ*L aliquot of serum was precipitated with 800 *μ*L of ice-cold methanol, vortexed for 10 s, and centrifuged at 15,000 × *g* for 10 min. The supernatant was divided into two portions, namely, 600 and 200 *μ*L for LC–MS and GC–MS analysis, respectively, which then were evaporated to dryness under a gentle nitrogen stream. For LC–MS analysis, the dried residue was reconstituted with 100 *μ*L of water: acetonitrile (80: 20 *v*/*v*) mixture, and a 10-*μ*L aliquot was used for LC–MS analysis. For GC–MS analysis, 50-*μ*L aliquot of MSTFA was added to the dried residue, mixed by vortexing for 10 s, and incubated at 70°C for 30 min. A 2-*μ*L aliquot of the derivatized sample was employed for GC–MS analysis.

### LC–MS analysis

LC analysis was carried out in an UltiMate 3000 HPLC system (Dionex Corporation, CA, USA). Metabolites were separated on a Luna-C18 (100 mm × 2.1 mm i.d., 3 *μ*m particle size) analytical column (Phenomenex, CA, USA) using a gradient procedure. Mobile phase A (A) was composed of 0.1% formic acid in ammonium acetate (5 mM) and mobile phase B (B) of 0.1% formic acid in acetonitrile. Mobile phase gradient was initiated at 5% B for 2 min, linearly increased to 80% B within 15 min, then increased to 95% B over 25 min, and maintained at 95% B for 5 min. MS analysis was performed with a micrOTOF-Q II quadrupole TOF mass spectrometer (Bruker Daltonik GmbH, Bremen, Germany). A standard electrospray ionization source was operated in a positive ionization mode. Spectra were collected in full scan mode from *m/z* 50–1500.

For MS/MS analysis, the experiment was performed with the same parameters in a multiple reaction monitoring mode at a rate of three spectra/s using an isolation width of ± *m/z* 4. Target ions were fragmented using nitrogen as collision gas and at a collision energy of 40 eV. *M/z* axis was calibrated using sodium formate (10 mM) at the end of every analysis run.

### GC–MS analysis

A 2-*μ*L aliquot of derivatized sample was introduced into a 7890A GC system equipped with a 5975C MSD (Agilent Technologies, Santa Clara, CA, USA). Metabolites were separated using a DB-5MS fused silica capillary column (15 m × 0.25 mm i.d., 0.25 *μ*m film thickness; Agilent J&W Scientific, Santa Clara, CA, USA). Injector temperature was set at 280°C. Carrier gas (helium) flow rate was at a constant 1 mL/min. Oven temperature was initially set at 80°C; after 1 min, temperature was raised to 200°C at 15°C/min and maintained for 3 min before increasing to 320°C at 20°C/min and maintained at this temperature for 7 min. Mass spectrometer was operated using an electron ionization mode at 70 eV with full scan acquisition over a range of *m/z* 40–550. Compounds corresponding to each peak on the GC–MS spectra were identified by comparing the sample MS spectra to those in the NIST 2011 mass spectral library and those of commercial standard compounds in our in-house database using an AMDIS software (National Institute of Standards and Technology (NIST), Gaithersburg, MD, USA). The amount of each metabolite was quantified using peak areas of quantifier ions and calculated by means of an MSD ChemStation software (Agilent Technologies).

### LC–MS data analysis

LC–MS data were converted into an mzXML file using a compassXport software (Bruker Daltonik GmbH). Raw data processing was performed by uploading mzXML data files to XCMS software [26] for feature detection and retention time alignment.

A set of XCMS processed data was subjected to multivariate data analysis employing a SIMCA 14 software package (Umetrics, Umeå, Sweden), which includes principal component analysis (PCA) and orthogonal projection to latent structure with discriminant analysis (OPLS-DA). OPLS-DA was carried out with unit variance scaling, which divides the weight of each variable by its standard deviation. Quality of metabolomics data was evaluated using relevant goodness of fit (*R*^2^) and predictability (*Q*^2^) values.

S-plot was performed to select potential biomarkers of an OVX condition. A protonated or adducted molecular ion of a metabolite feature selected from S-plot was exported for metabolite search to an online database (METLIN, HMDB and lipid maps) with a mass tolerance of ± 30 ppm. Additionally, heat map was constructed with a MetaboAnalyst [27] web-based tool.

A flow chart of the GC–and LC–MS protocols is shown in Fig 1B.

### Statistical analysis

All data are expressed as mean ± standard error of mean (SEM). Differences between groups were analyzed using one-way analysis of variance, followed by Tukey–Kramer post hoc test. Unpaired Student’s t-test was employed for comparison between means of two groups. Values are considered significantly different at *p*-value <0.05. Data were analyzed using a GraphPad Prism 5.0 software package (GraphPad Software, San Diego, CA, USA).

## Results

### Body and uterine weights

In the present study, rat body weights were recorded weekly during the course of the experiment. All rats, particularly OVX rats, progressively gained body weights throughout the course of the experiment. At week 12 post-treatment, OVX rat body weight increased to 355.9 ± 11.5 g, significantly higher compared to 277.9 ± 12.4 g of SHAM rat (*p*-value <0.01), while treatment with DPHD and *C*. *comosa* extract suppressed OVX rat body weight to 306.8 ± 5.3 and 291.0 ± 7.3 g, respectively (S1 Table in S1 File).

Uterotrophic effect is one of the important biological actions of estrogen on target tissues, so atrophy of the uterus provides an evidence for successful ovariectomy in OVX rats. Uterine weights of all animals were determined at sacrifice, those of OVX rats (0.51 ± 0.10 g/kg BW) being 50% of SHAM (1.03 ± 0.04 g/kg BW). Treatment with DPHD and *C*. *comosa* extract increased uterine weight of the OVX rats to 1.32 ± 0.30 and 0.92 ± 0.10 g/kg BW, respectively, similar to that of SHAM (S1 Table in S1 File).

### Multivariate data analysis of LC–MS untargeted serum metabolomics profiles

**Fig 2:**
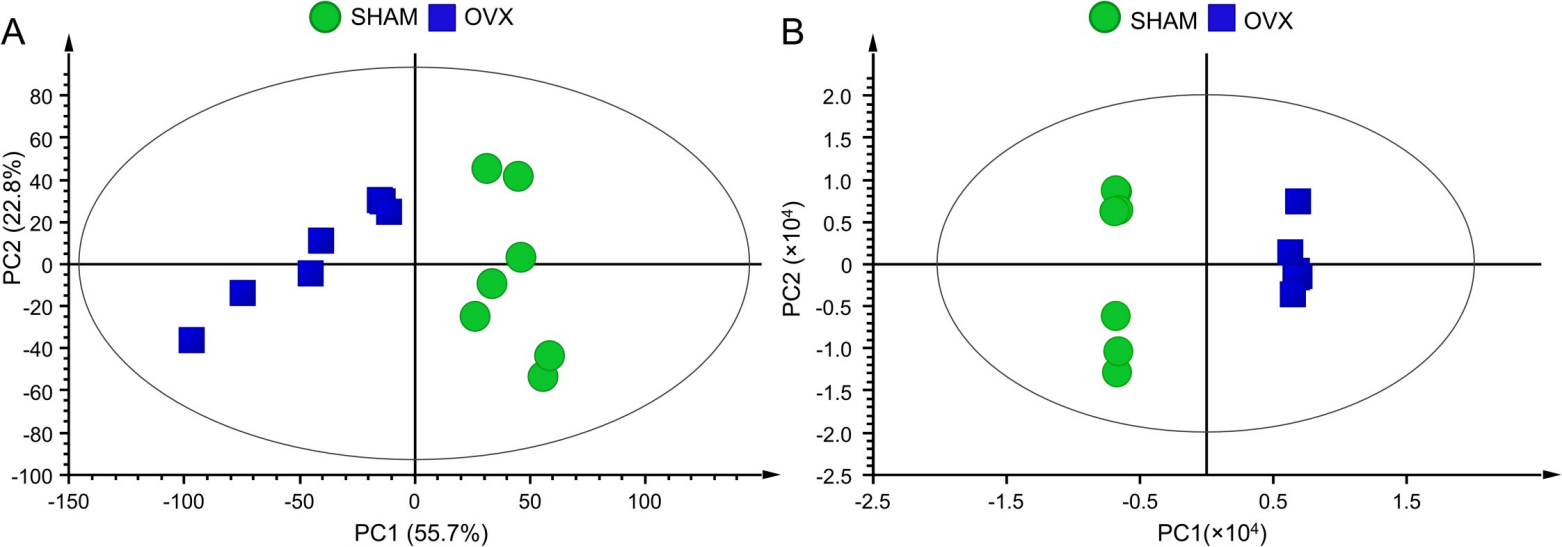
Multivariate data analysis of untargeted metabolomics profiles. (A) Principal component analysis. (B) Orthogonal projection to latent structure with discriminant analysis. Data were obtained from LC–MS-based untargeted metabolomics analysis of serum samples of sham-operated (SHAM) (green circles) and bilateral ovariectomized (OVX) (blue squares) rats at week 12 post-treatment (*n* = 7 per group). PC, principal component.

Metabolomics analysis revealed changes of several endogenous metabolites in sera of OVX compared to SHAM rat group. Using LC–MS in the positive ion mode, 8,750 variables were detected in the metabolomics datasets. PCA score plot showed distinct separations of serum metabolites obtained from SHAM and OVX rats (Fig 2A). In order to enhance intergroup differences, we also conducted a supervised OPLS-DA using unit variance scaled LC–MS data and class information (sample group) as X- and Y-variable, respectively, which revealed clustering of different sample groups with *R*^2^ and *Q*^2^ values of 0.999 and 0.875, respectively, indicating 21.8% predictive variations with one component and 60.9% orthogonal variations with six components (Fig 2B).

### Identification of OVX-associated serum metabolites

**Fig 3:**
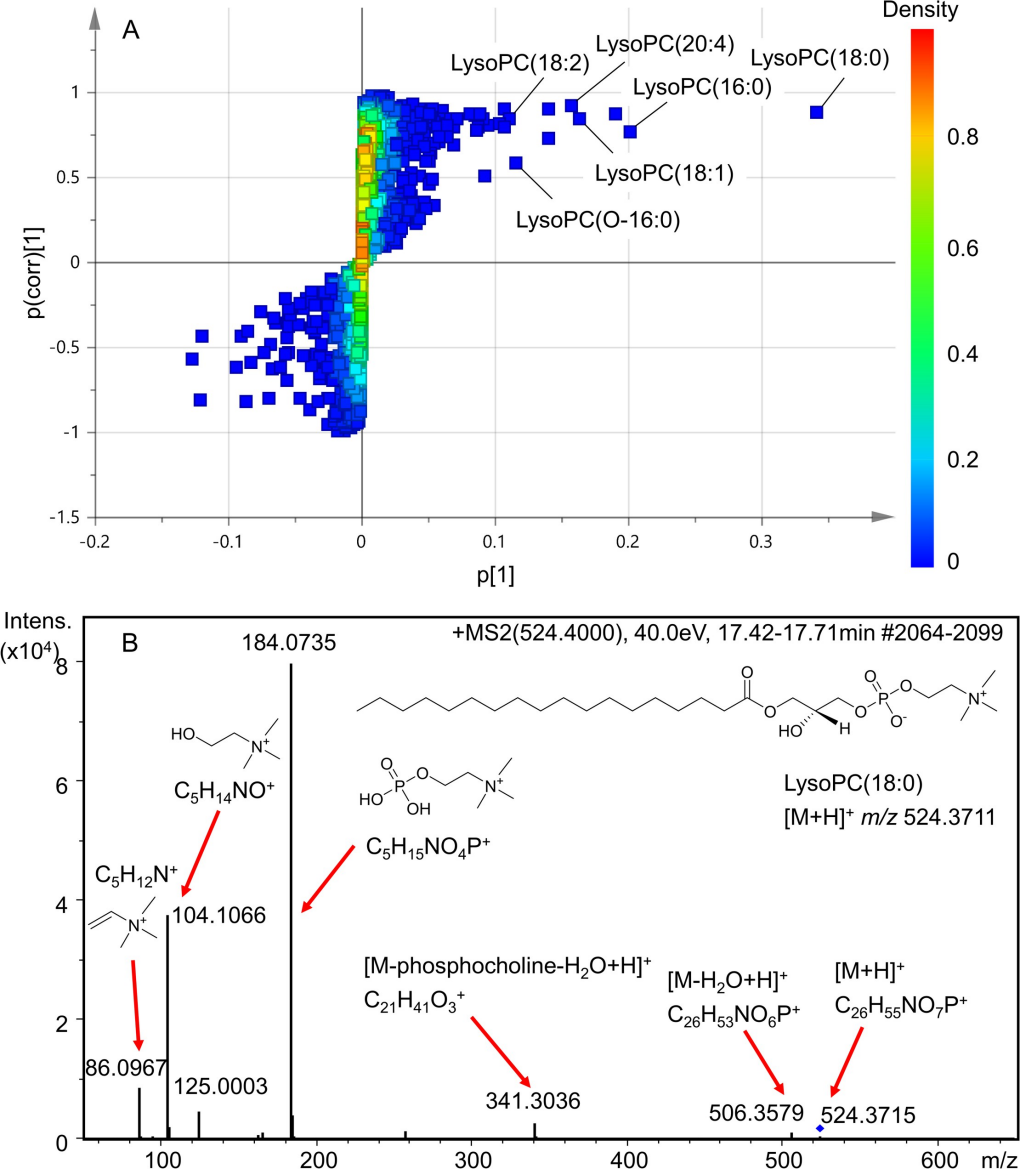
Identification of lysophosphatidylcholines (lysoPCs) as OVX-associated metabolites. (A) S-loading plot of serum metabolic profiles in orthogonal projection to latent structure with discriminant analysis (OPLS-DA). Each square represents a metabolite feature. The upper right and lower left quadrant contains metabolite features with increased and decreased levels in OVX compared to SHAM samples, respectively. Heat scale (Density) indicates *Z-score*, ranging from highest (red) to lowest (blue). p(corr)[1], reliability; p[1], magnitude. (B) LC–MS/MS spectrum of protonated lysoPC (18:0) fragment ions at 40 eV. Ions of *m/z* 86.0967, 104.1066, and 184.0735 arose from the phosphocholine head group, and ions of *m/z* 341.3036 and 506.3579 arose from the loss of water or phosphocholine from the protonated lysoPC molecular ion of *m/z* 524.3715 (blue diamond).

In order to investigate putative serum metabolite biomarkers associated with ovariectomy, pairwise comparative OPLS-DA between SHAM and OVX rats was further explored with an S-loading plot, which reveals changes in magnitude (p[1]) and reliability (p(corr)[1]) of each metabolite ion. Cutoff is set at p[1] >0.1 and p(corr)[1] >0.5 for markers with increased levels and at p[1] <−0.1 and p(corr)[1] <−0.5 for markers with decreased levels in OVX samples compared to SHAM. Using these criteria, 12 and 2 metabolites were shown to be elevated and reduced, respectively, in OVX samples (Fig 3A).

By searching exact masses of these metabolites against those in online databases, the majority of metabolites increased in OVX samples belonged to lysophosphatidylcholines (lysoPCs), specifically, 1-stearoyl-glycero-3-phosphocholine [lysoPC (18:0)], 1-arachidonoyl-glycero-3-phosphocholine [lysoPC (20:4)], 1-oleoyl-glycero-3-phosphocholine [lysoPC (18:1)], 1-linoleoyl-glycero-3-phosphocholine [lysoPC (18:2)], 1-palmitoyl-glycero-3-phosphocholine [lysoPC (16:0)], and 1-hexadecyl-glycero-3-phosphocholine [lysoPC (O-16:0)] (Fig 3A). Among them, lysoPC (18:0) had the highest p[1] and p(corr)[1] values of 0.34 and 0.89, respectively, indicating the greatest difference in level and highest reliability between OVX and SHAM samples.

As lysoPCs could serve as possible biomarkers of metabolic disturbances in a post-menopausal condition, their chemical structures were further confirmed by LC–MS/MS analysis, which showed similar spectra patterns indicative of phosphocholine as head group ion (Fig 3B and S3 Fig in S1 File). For example, the MS/MS fragment spectrum of lysoPC (18:0), which had the most change in level among all identified metabolites, demonstrated *m/z* 86.0967 (C_5_H_12_N^+^, [choline−H_2_O]^+^), 104.1066 (C_5_H_14_NO^+^, [choline]^+^), and 184.0735 (C_5_H_15_NO_4_P^+^, [phosphocholine]^+^) (Fig 3B). Additionally, the loss of phosphocholine or a water molecule from lysoPCs was commonly observed, with, for instance, *m/z* 341.3036 (C_21_H_41_O_3_^+^) arising from the loss of phosphocholine and a water molecule, and *m/z* 506.3579 (C_26_H_53_NO_6_P^+^) from a loss of water from lysoPC (18:0). Together, the fragment ions provided strong evidence for the structural characterization of the changed ions as lysoPCs.

**Fig 4:**
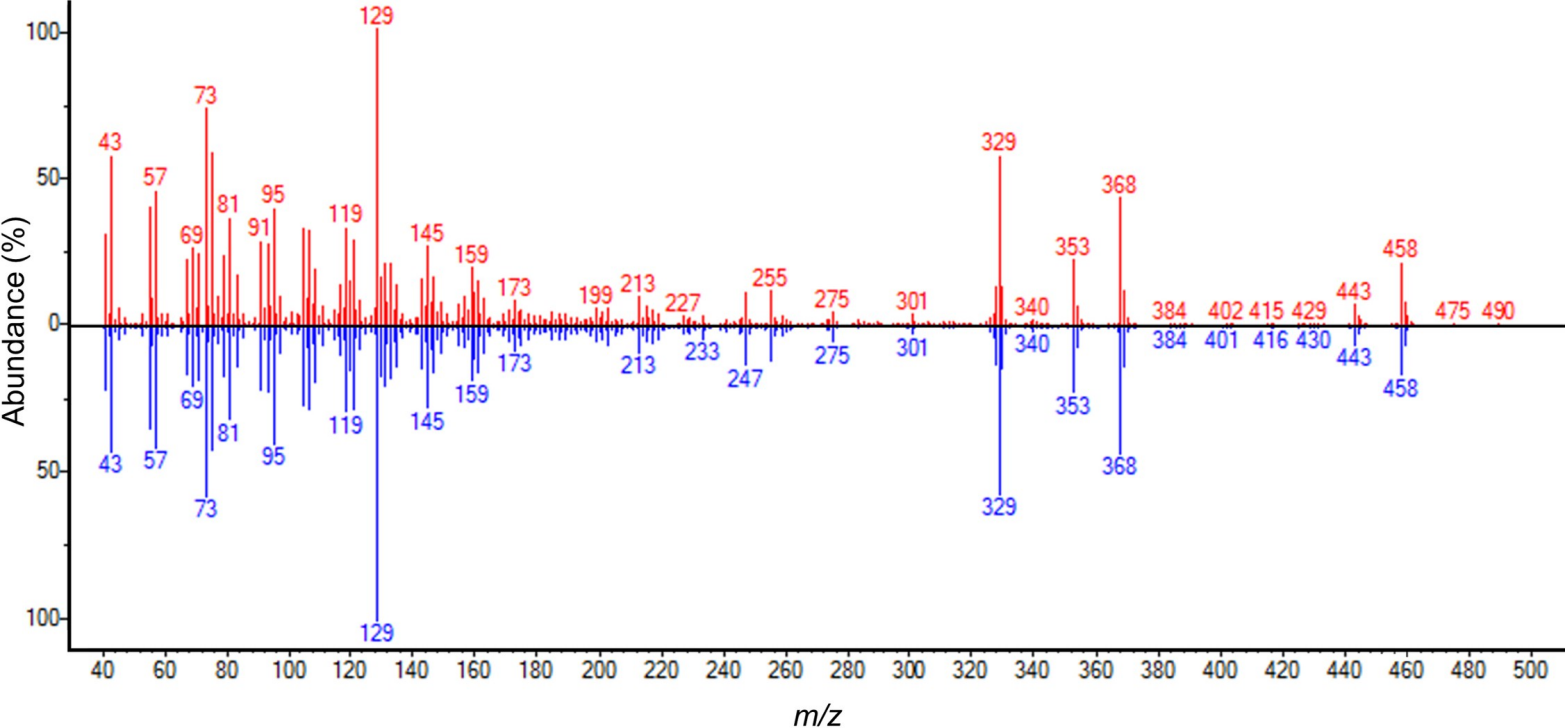
Representative GC–MS spectrum of trimethylsilyl-derivatized cholesterol. Red, MS spectrum of trimethylsilyl-derivatized cholesterol from serum of ovariectomized rat; blue, spectrum from GC–MS NIST library.

In order to further expand the coverage of metabolite classes in our studies, GC–MS-based metabolomics analyses of 47 metabolites in five metabolite classes (amino acid, carbohydrate, fatty acid, lipid, and organic acid) were conducted using GC–MS, followed by interpretation of spectra by comparing with those in NIST or in-house libraries using an AMDIS software (Fig 4). Based on peak area ratios of OVX: SHAM samples, metabolites significantly elevated in OVX were 1,5-anhydrosorbitol, arachidonic acid, beta-sitosterol, campesterol, cholesterol, galactopyranose, glucose, glycerol, linoleic acid, mannopyranose, palmitic acid, ribopyranose, stearic acid, and talopyranose (Table 1). These altered metabolites, along with lysoPCs identified from LC–MS analysis, could serve as potential metabolite biomarkers for estrogen deficiency condition.

**Table 1:** GC–MS retention time, quantifier and qualifier ions, and relative peak area of metabolites in ovariectomized (OVX) compared to sham-operated (SHAM) rat sera.

Metabolite	Retention time	Ion		Fold change
	(min)	Quantifier	Qualifier	(OVX/SHAM)
Lactic acid	2.51	219	117	1.4
2-Propenoic acid	2.53	147	191	1.6
Alanine	2.86	116	100	1.8
Glycine	3.03	102	176	2.1
2-Hydroxybutyric acid	3.07	131	147	1.2
3-Hydroxybutyric acid	3.37	147	191	1.4
Valine	3.86	144	218	1.4
4-Hydroxybutyric acid	4.16	233	204	0.9
Urea	4.18	189	171	1.5
Leucine	4.37	158	102	1.3
Glycerol	4.39	147	205	2.3**
Isoleucine	4.56	158	218	1.4
Proline	4.60	142	216	1.1
Succinic acid	4.76	247	172	1.8
Pyrimidine	4.96	80	53	1.6
Serine	5.16	100	218	1.4
Threonine	5.38	117	218	1.5
Methionine	6.49	176	128	1.4
Creatinine	6.76	115	257	1.5
Phenylalanine	7.33	218	192	1.5
Arabinopyranose	7.55	217	205	2.1
Ribopyranose	7.65	217	205	1.8*
Mannopyranose	7.95	217	205	1.9**
Talopyranose	8.94	217	205	1.9**
1,5-Anhydrosorbitol	9.25	147	217	1.9**
Galactopyranose	9.28	129	191	2.5**
Methyl palmitate	9.57	87	143	1.3
Tyrosine	9.64	218	100	1.9
Methyl stearate	11.86	87	143	1.5
Glucose	10.06	319	217	2.3**
Hexadecenoic acid	10.51	311	145	1.7
Palmitic acid	10.77	313	145	1.6*
Myo-inositol	11.23	305	318	1.8
Tryptophan	12.85	202	291	1.7
Linoleic acid	12.92	337	145	1.7*
Oleic acid	12.99	339	145	1.4
Stearic acid	13.27	341	145	1.8*
Methyl arachidonate	13.36	79	91	1.6
Arachidonic acid	14.26	117	129	1.7**
1-Monomyristin	14.39	343	117	0.9
2-Monopalmitin	15.31	218	117	1.6
1-Monopalmitin	15.47	371	117	1.5
2-Monostearin	16.20	218	117	1.8
1-Monostearin	16.35	487	117	1.6
Cholesterol	17.92	368	458	2.0**
Campesterol	18.14	472	343	2.5**
Beta-sitosterol	18.48	486	396	2.6**

* *p*-value <0.05

** *p*-value <0.01 compared to SHAM.

### Protective effect of DPHD and *C*. *comosa* ethanol extract on metabolic disturbance of OVX rat

**Fig 5:**
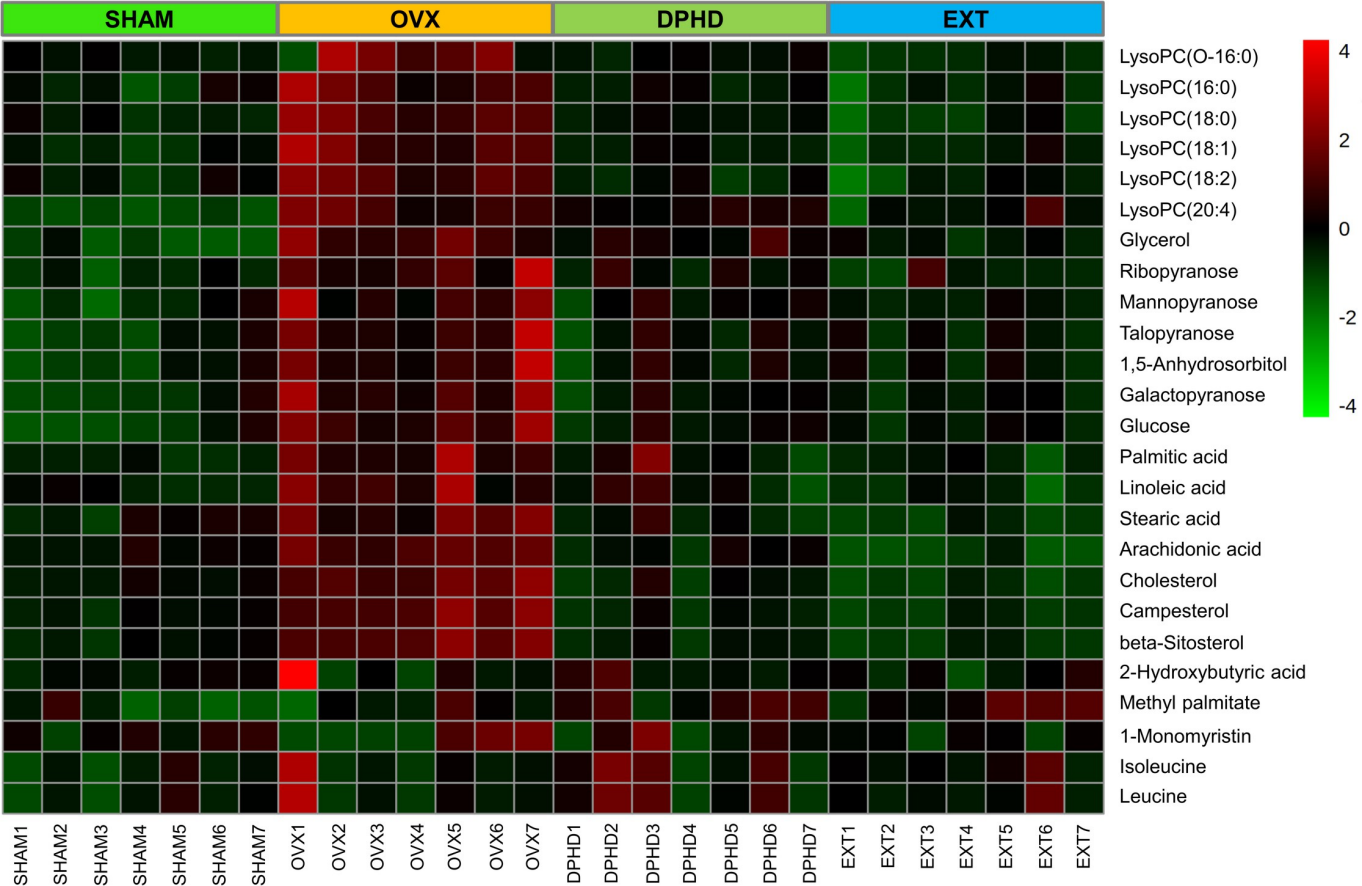
Heat map of rat serum metabolite levels. Rat serum metabolites were quantified by LC–and GC–MS-based metabolomics analysis. Each square represents serum data from each sham-operated (SHAM), bilateral ovariectomized (OVX), OVX treated with diarylheptanoid (3*R*)-1,7-diphenyl-(4*E*,6*E*)-4,6-heptadien-3-ol (DPHD), and OVX treated with *Curcuma comosa* ethanol extract (EXT) rat (*n* = 7 per group). *Z-score* scale ranges from highest (red) to lowest (green) abundance level.

**Fig 6:**
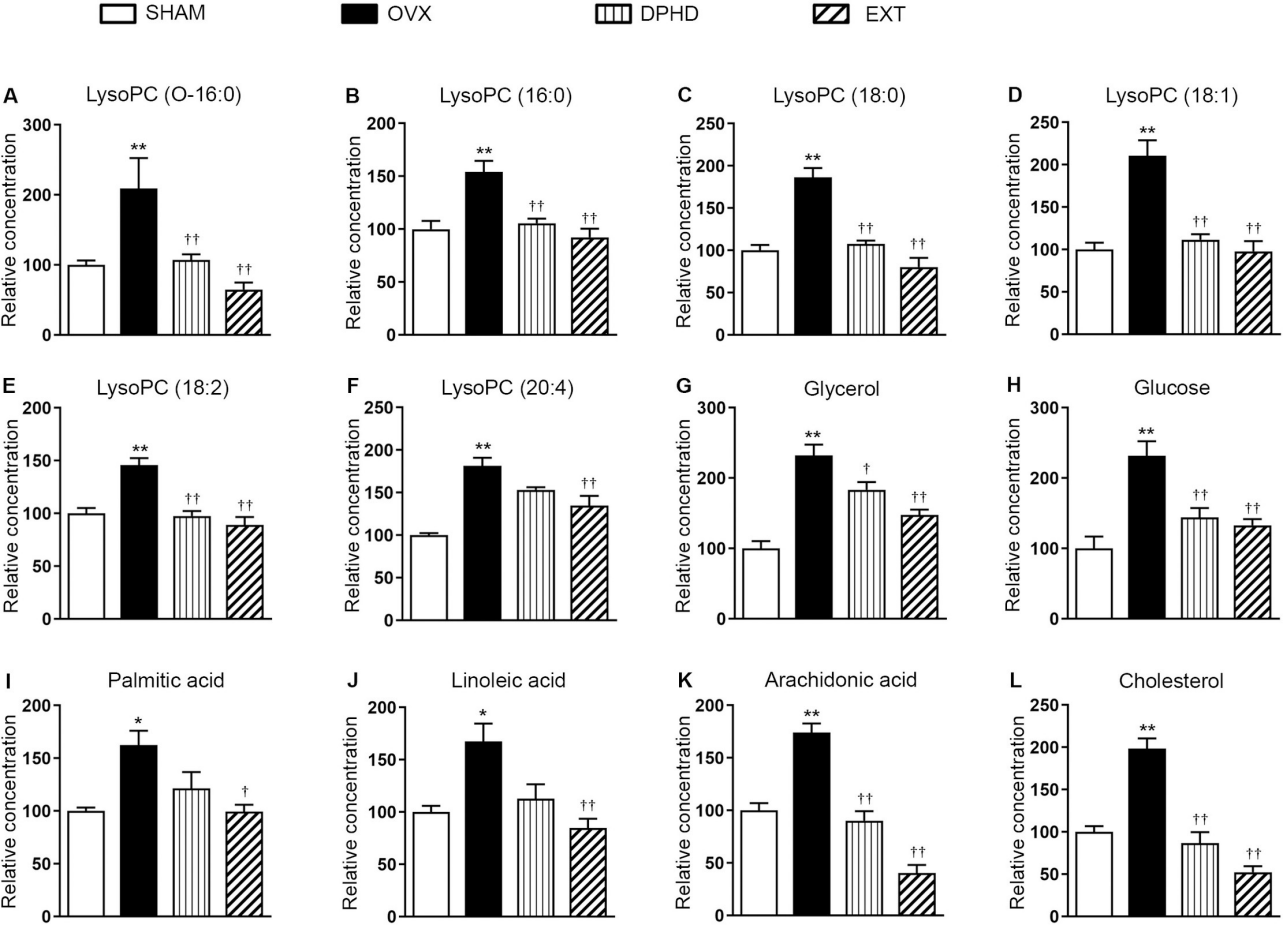
Relative levels of potential serum biomarkers of metabolic changes arising from ovariectomy. Rat serum metabolites were quantified by LC–and GC–MS-based metabolomics analysis. Results are shown as mean ± SEM (*n* = 7) metabolite peak area (concentration) relative to sham-operated rat (SHAM) of bilateral ovariectomized (OVX), OVX treated with diarylheptanoid (3*R*)-1,7-diphenyl-(4*E*,6*E*)-4,6-heptadien-3-ol (DPHD), and OVX treated with *Curcuma comosa* ethanol extract (EXT) rat. *p-value <0.05, **p-value <0.01 compared to SHAM. ^†^*p*-value <0.05, ^††^ *p*-value <0.01 compared to OVX.

From the results of metabolomics analysis of OVX and SHAM rat sera, biomarkers identified by both GC–and LC–MS techniques were subsequently used to evaluate the therapeutic efficacy of DPHD and *C*. *comosa* ethanol extract in OVX rats (DPHD and EXT, respectively). A heat map was generated to demonstrate the effects of each treatment on levels of putative metabolite biomarkers and five other metabolites that did not show differences between OVX and SHAM rats (controls). Levels of the putative metabolites in EXT were more similar to those in SHAM compared to untreated OVX group, indicating the effectiveness of *C*. *comosa* extract treatment (Fig 5). This was confirmed when the average level of each putative metabolite biomarker (lysoPCs, glycerol, glucose, palmitic acid, linoleic acid, arachidonic acid, and cholesterol) was compared among the test groups (Fig 6). Interestingly, DPHD treatment could only reverse some of the putative biomarkers in OVX to SHAM levels; It did not significantly lower the elevated levels of lysoPC (20:4), palmitic acid, and linoleic acid. The findings indicated the potential of DPHD and *C*. *comosa* ethanol extract for treating metabolic changes induced by ovariectomy.

## Discussion

The study employed LC–and GC–MS techniques for analysis of serum metabolites in SHAM, OVX, DPHD, and EXT rats, which revealed that ovariectomy over a long period (12 weeks) resulted in hyperlipidemia and significant increase in levels of proinflammatory mediators, lysoPCs and AA, compared to sham operation. Treatment of OVX rat with DPHD and *C*. *comosa* ethanol extract reverted the changes to SHAM levels, with the extract demonstrating a more effective effect. Currently, the involvement of lysophospholipids signaling in physiology and pathogenesis has been widely investigated. Their activities are mediated by specific G protein-coupled receptors, which are implicated in several human diseases [28, 29]. Intervention of lysophospholipids signaling process plays a key medical role in providing therapeutic means for treatment of such diseases. Our study is the first report to show the effectiveness of *C*. *comosa* treatment in countering the rise of serum lysoPCs and AA in OVX rat.

LysoPCs are primarily generated from hydrolysis of phosphatidylcholine at *sn*-2 position by phospholipase A2 (PLA2) [30] generating free fatty acids, such as AA, which are then converted to potent proinflammatory eicosanoid mediators, namely, prostaglandins, thromboxanes, leukotrienes, and lipoxins [31]. LysoPC can be further hydrolyzed to lysophosphatidic acid (LPA) by autotaxin (ATX), an ectonucleotide pyrophosphatase/ phosphodiesterase 2, which has lysophospholipase D activity [32]. These enzymatic activities have been suggested to act as endogenous atherogenic factors generating LPA during mild conditions of LDL oxidation, during which LPA accumulates in the lipid core of atherosclerotic plaque [33]. Additionally, alterations in the ATX-LPA pathway have been implicated in metabolic and inflammatory disorders, such as obesity, insulin resistance, metabolic syndrome, cardiovascular disease, neurogenesis, and cancer [28, 29]. LysoPCs can also activate multiple signaling pathways involved in oxidative stress and inflammatory responses by increasing synthesis of cyclooxygenase-2 and a number of inflammatory cytokines that further stimulate ATX production [34]. Activation of PLA2 has been reported in inflammatory processes in animal models of OVX-induced estrogen deficiency [35, 36]. Levels of lysoPCs are increased in post-menopausal women [37].

In mice, hyperlipidemia is implicated in contributing to circulating LPA [38]. Several reports have shown the role of adipocyte ATX-LPA axis in obesity and pathogenesis of related diseases [39–41]. Adipocyte-derived ATX constitutes almost half of plasma LPA [42], and the expression and release of ATX from adipocytes activate preadipocyte proliferation [43]. ATX expression is highly upregulated during adipocyte differentiation in obese and diabetic db/db mice [44], suggesting the role of ATX in regulating adipose tissue development and obesity-associated pathologies [43, 45, 46]. Recently, an essential role of adipose tissue ATX in breast cancer progression has been reported [28]. The high amounts of secreted ATX in breast cancer are derived from adjacent adipose-rich tissues, while breast cancer cells themselves have relatively low ATX activity [28]. In our study, OVX rat showed hyperlipidemia and greater visceral adipose tissue mass with larger adipocyte size similar to a previous study [18]. Thus, it can be surmised that dysregulated adipose tissue mass in OVX rat forms, in part, a source of circulating lysoPCs and AA.

Using LC–MS-based technique, we identified significant increases in OVX rat serum of lysoPC (16:0), lysoPC (18:0), lysoPC (18:1), lysoPC (18:2), and lysoPC (20:4), phospholipids more hydrophilic than the ceramides, ceramide-1-phosphate, and sphingomyelins previously reported [24]. Thus, the findings in this work have added to the list of metabolites observed to be increased in OVX rat serum, thereby providing a more comprehensive picture of the metabolic disturbances caused by ovariectomy.

Elevation of lysoPCs, particularly lysoPC (18:0), indicates the existence of oxidant stress in OVX rat. Overproduction of reactive oxygen species could damage cellular lipids and proteins leading to cellular dysfunction. Reversion to SHAM levels of lysoPCs by *C*. *comosa* extract indicated its ability to attenuate oxidant stress in the whole animal, consistent with previous reports of DPHD and *C*. *comosa* extract protecting cells from oxidant damage [47–49].

Increase in serum level of AA, a metabolite associated with inflammatory process related to enhanced fatty acid biosynthesis in OVX rat, was in agreement with previous reports [6, 8]. We also observed increased levels of several fatty acids, such as palmitic acid, which can induce inflammatory responses [50, 51] and might be related to the elevated level of AA. Previous studies have shown the increase in plasma lysoPCs, namely, palmitoyl (C16:0) and stearoyl (C18:0) acyl chains, greatly enhances AA release from membranes [52, 53]. Elevated levels of cholesterol, glucose, and glycerol in OVX rat have been observed in previous metabolomics studies conducted in rats with OVX-induced obesity and bone loss [5, 6]. In the present study, treatment of OVX rats with DPHD or *C*. *comosa* extract for 12 weeks returned levels of these metabolites to those of SHAM rats.

In addition to estrogenic-like activities, an anti-inflammatory effect of *C*. *comosa* extract has been reported in diet-induced hypercholesterolemia rabbit by decreasing production of several inflammatory cytokines, such as IL-1 and TNF-α, and of atherosclerotic plaque [21, 22]. Targeting the regulation of lysoPCs and AA production could be a promising therapeutic intervention in OVX-associated metabolic disturbances. Although the amount of DPHD used was similar to that present in *C*. *comosa* ethanol extract, it is worth noting that the ameliorating effects of *C*. *comosa* ethanol extract was superior to DPHD, suggesting the crude extract contains other bioactive compounds. Ethanol extract of *C*. *comosa* contains both nonphenolic and phenolic diarylheptanoids [12, 13, 24]. DPHD used in the present study is the major nonphenolic diarylheptanoid in the extract having the highest estrogenic-like activity [14]. However, *C*. *comosa* ethanol extract also contains others diarylheptanoids, such as phenolic diarylheptanoids, (3*S*)-1-(3,4-dihydroxyphenyl)-3-hydroxy-7-phenyl-(6*E*)-6-heptene and (3*R*)-1-(4-hydroxyphenyl)-7-phenyl-(6*E*)-6-hepten-3-ol, with high antioxidant and anti-inflammation activities [12, 23, 48]. In addition, it is highly likely that other bioactive diarylheptanoids (as well as compounds of different chemical scaffolds) exist in *C*. *comosa* and their presence in the ethanol extract could participate (synergistically or antagonistically) in influencing metabolic conditions of estrogen deficiency. However, the roles of those metabolites, particularly lysoPCs, should be further explored and validated for their promising ability to serve as potential biomarkers of post-menopausal metabolic syndromes.

The major goal of the present study was to advance our understanding of the effects of traditional use of *C*. *comosa* extract as a dietary supplement for health promotion in post-menopausal women. Hence, to mimic the use in post-menopausal women, *C*. *comosa* extract was intragastrically administered to OVX rats, and so were SHAM and OVX control rats, which similarly received vehicle of *C*. *comosa* extract intragastrically. However, due to the limited availability of purified DPHD, this compound was subcutaneously administered to OVX rats. The differences in the vehicle and route of administration between DPHD and the rest of treatment groups should be noted. It is, therefore, a limitation of the study with DPHD, and interpretation of such data in comparison with other treatment groups warrants careful attention.

## Conclusions

In this study, we employed LC–MS and GC–MS techniques for analyses of methanol-extracted metabolites from sera of all test rats. This approach revealed that rats over a relatively long period of ovariectomy had elevated levels of proinflammatory mediators, lysophosphatidylcholines (lysoPCs) and arachidonic acid (AA), amounts of which were restored to those of sham-operated rats by treatment with ethanol extract of the herbal plant, *Curcuma comosa*, traditionally used to treat maladies of post-menopausal women. Moreover, this reversion in proinflammatory mediators levels could be achieved with (3*R*)-1,7-diphenyl-(4*E*,6*E*)-4,6-heptadien-3-ol, the principal component of *C*. *comosa* ethanol extract. These altered levels of lysoPCs and AA may serve as potential biomarkers for inflammatory disturbances in post-menopausal women, which could be ameliorated by judicial application of traditional herbal remedies.

## Supporting information

S1 File S1 Table and S1–S3 Figs. S1 Table. Effects of *Curcuma comosa* treatment on rat body and uterine weights. S1 Fig. GC–MS total ion chromatogram of serum metabolites. S2 Fig. LC–MS total ion chromatogram of rat serum metabolites. S3 Fig. MS/MS spectra of lysophosphatidylcholines (lysoPCs).
(PDF)
