# Supplementary material for: Lowering of lysophosphatidylcholines in ovariectomized rats by Curcuma comosa
Source: PLoS One. 2022 May 19;17(5):e0268179. doi: 10.1371/journal.pone.0268179 (PMC9119514; doi:10.1371/journal.pone.0268179)
Supplement: S1 File — S1 Table. Effects of Curcuma comosa treatment on rat body and uterine weights. S1 Fig. GC–MS total ion chromatogram of serum metabolites. S2 Fig. LC–MS total ion chromatogram of rat serum metabolites. S3 Fig. MS/MS spectra of lysophosphatidylcholines (lysoPCs). (PDF) [file pone.0268179.s001.pdf]

# Supporting Information

## Lowering of lysophosphatidylcholines in ovariectomized rats by *Curcuma comosa*

Jetjamnong Sueajai[1,2], Nareerat Sutjarit[3], Nittaya Boonmuen[4], Saranya Auparakkitanon[2], Nantida Noumjad[2], Apichart Suksamrarn[5], Nawaporn Vinayavekhin[6,7*], Pawinee Piyachaturawat[4]

[1] Toxicology Graduate Program, Faculty of Science, Mahidol University, Bangkok 10400, Thailand

[2] Department of Pathology, Faculty of Medicine Ramathibodi Hospital, Mahidol University, Bangkok 10400, Thailand

[3] Graduate Program in Nutrition, Faculty of Medicine, Ramathibodi Hospital, Mahidol University, Bangkok 10400, Thailand

[4] Department of Physiology, Faculty of Science, Mahidol University, Bangkok 10400, Thailand

[5] Department of Chemistry, Faculty of Science, Ramkhamhaeng University, Bangkok 10240, Thailand

[6] Center of Excellence in Natural Products Chemistry, Department of Chemistry, Faculty of Science, Chulalongkorn University, Bangkok 10330, Thailand

[7] Center of Excellence in Biocatalyst and Sustainable Biotechnology, Faculty of Science, Chulalongkorn University, Bangkok 10330, Thailand

* Corresponding author

E-mail: nawaporn.v@chula.ac.th (NV)

**S1 Table. Effects of *Curcuma comosa* treatment on rat body and uterine weights.**

| Treatment | Body weight (g) | | | Uterine weight (g/kg BW) |
|---|---|---|---|---|
| | Initial | Final | Change | |
| SHAM | 203.1 ± 3.6 | 277.9 ± 12.4 | +74.8 ± 9.8 | 1.03 ± 0.04 |
| OVX | 210.4 ± 4.9 | 355.9 ± 11.5** | +145.4 ± 11.0* | 0.51 ± 0.10* |
| DPHD | 207.3 ± 3.8 | 306.8 ± 5.3†† | +99.5 ± 1.9†† | 1.32 ± 0.30†† |
| EXT | 214.1 ± 4.4 | 291.0 ± 7.3†† | +76.9 ± 8.2†† | 0.92 ± 0.10† |

Rats were randomly assigned as sham-operated (SHAM) or ovariectomized (OVX). OVX rats were treated with *C. comosa* ethanol extract (EXT) (500 mg/kg BW, intragastric) or (3*R*)-1,7-diphenyl-(4*E*,6*E*)-4,6-heptadien-3-ol (DPHD) (50 mg/kg BW, subcutaneous) for 12 weeks. Result is reported as mean ± SEM ($n$ = 7). **p*-value <0.01, significantly different compared to SHAM. †*p*-value <0.05, ††*p*-value <0.01, significantly different compared to OVX.

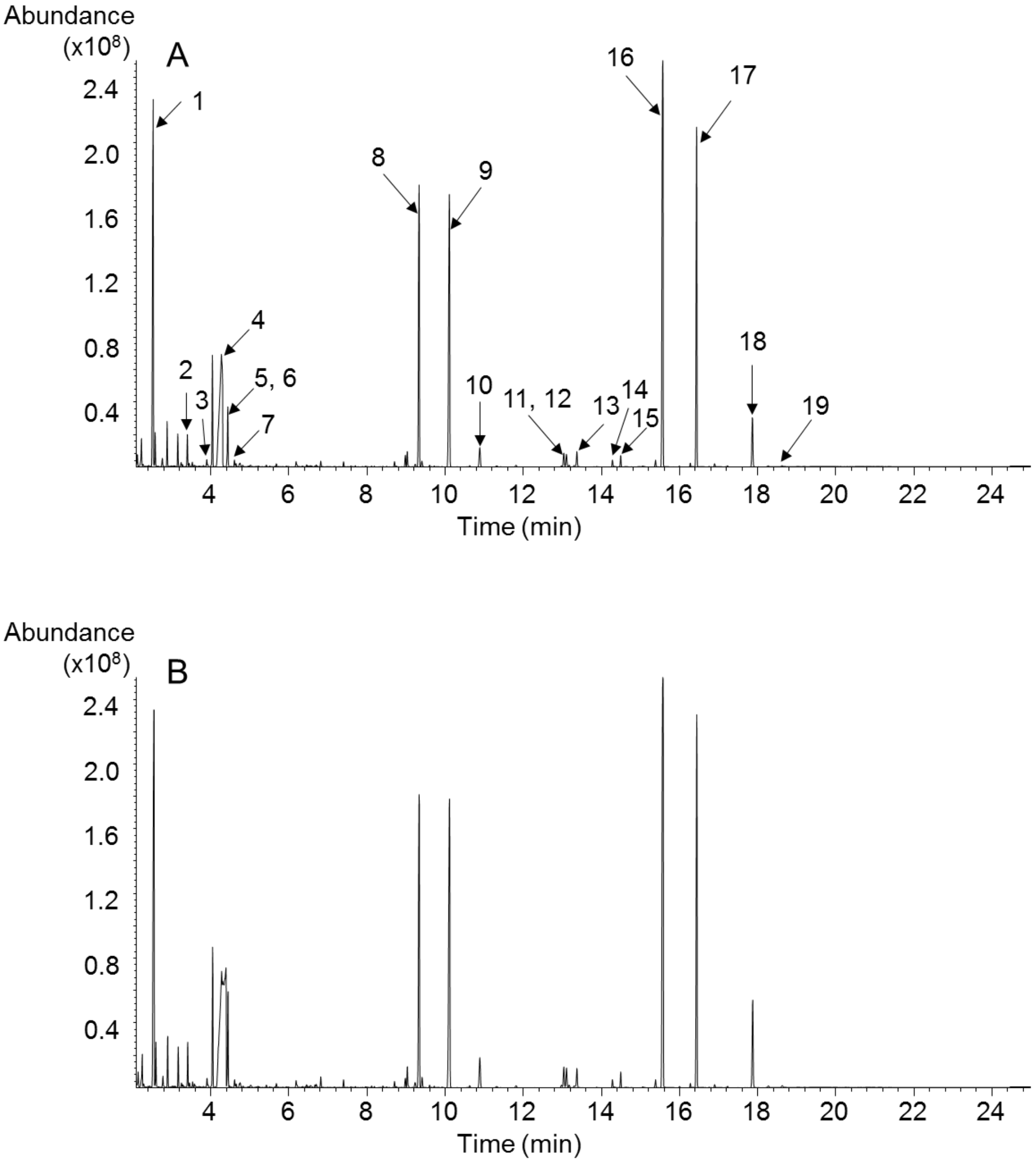


**S1 Fig. GC–MS total ion chromatogram of serum metabolites.** (A) Sham-operated (SHAM) rat. (B) Ovariectomized (OVX) rat. Metabolites were separated on a DB-5MS fused silica capillary column (15 m × 0.25 mm i.d., 0.25 $\mu$m film thickness), with injector temperature set at 280°C, carrier gas (helium) flow rate at 1 mL/min, and oven temperature initially at 80°C and after 1 min raised to 200°C at 15°C/min, maintained for 3 min before increasing to 320°C at 20°C/min and maintained at this temperature for 7 min. Mass spectrometer was operated using an electron ionization mode at 70 eV with full scan acquisition of *m/z* 40–550. (1) Lactic acid, (2) 3-Hydroxybutyric acid, (3) Valine, (4) Urea, (5) Leucine, (6) Glycerol, (7) Isoleucine, (8) Mannopyranose, (9) Glucose, (10) Palmitic acid, (11) Linoleic acid, (12) Oleic acid, (13) Stearic acid, (14) Arachidonic acid, (15) 1-Monomyristin, (16) 1-Monopalmitin, (17) 1-Monostearin, (18) Cholesterol, (19) Beta-sitosterol.

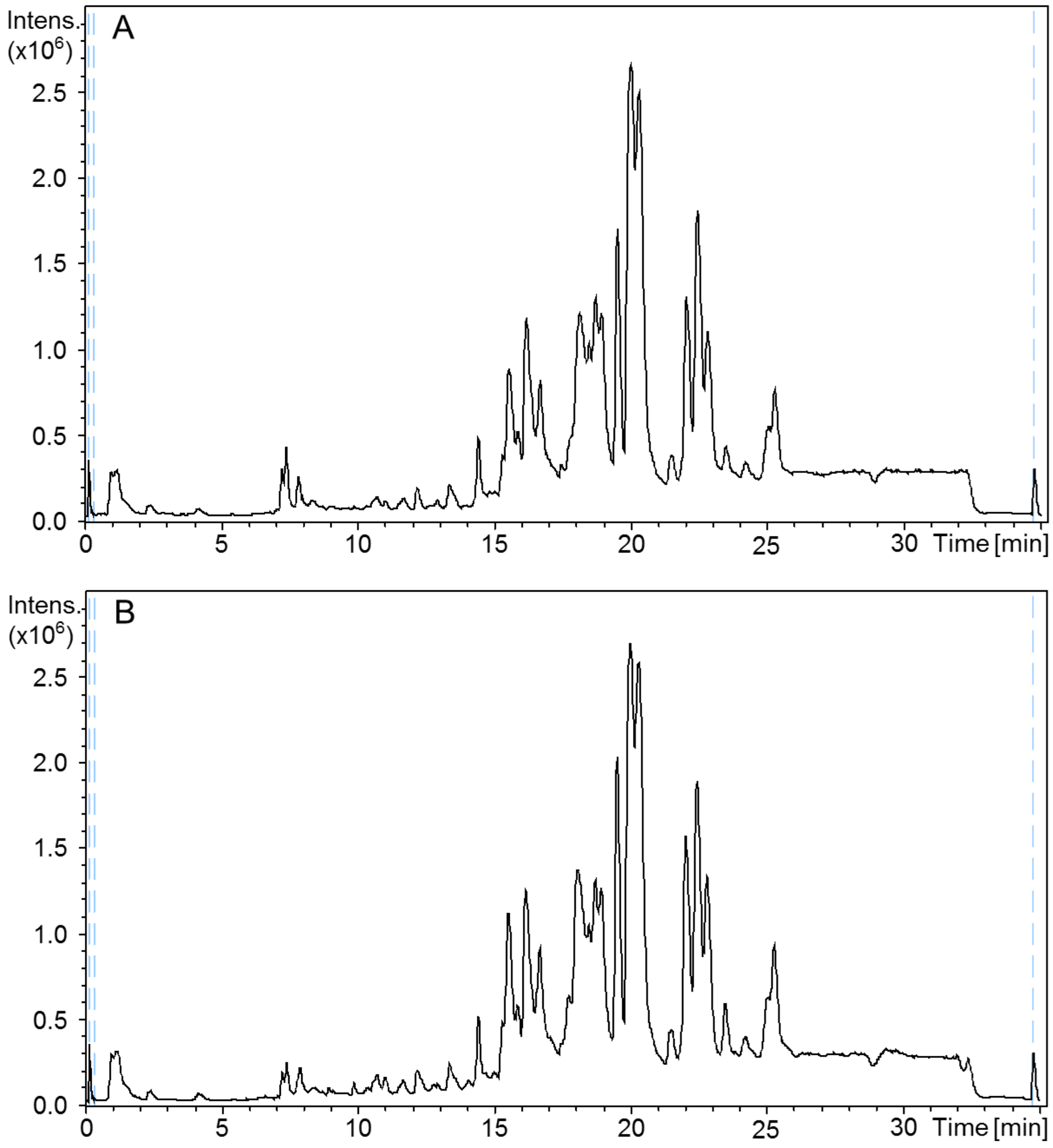


**S2 Fig. LC–MS total ion chromatogram of rat serum metabolites.** (A) Sham-operated (SHAM) rat. (B) Ovariectomized (OVX) rat. A gradient procedure comprised of a mobile phase A (0.1% formic acid in 5 mM ammonium acetate) and mobile phase B (0.1% formic acid in acetonitrile). Mobile phase gradient was initiated at 5% B for 2 min, linearly increased to 80% B within 15 min, then increased to 95% B over 25 min, and maintained at 95% B for 5 min. MS analysis was performed using a standard electrospray ionization source operated in a positive ionization mode, and spectra were collected in full scan mode from *m/z* 50–1500. Lysophosphatidylcholines were eluted between 15–20 min.

A LysoPC(O-16:0)

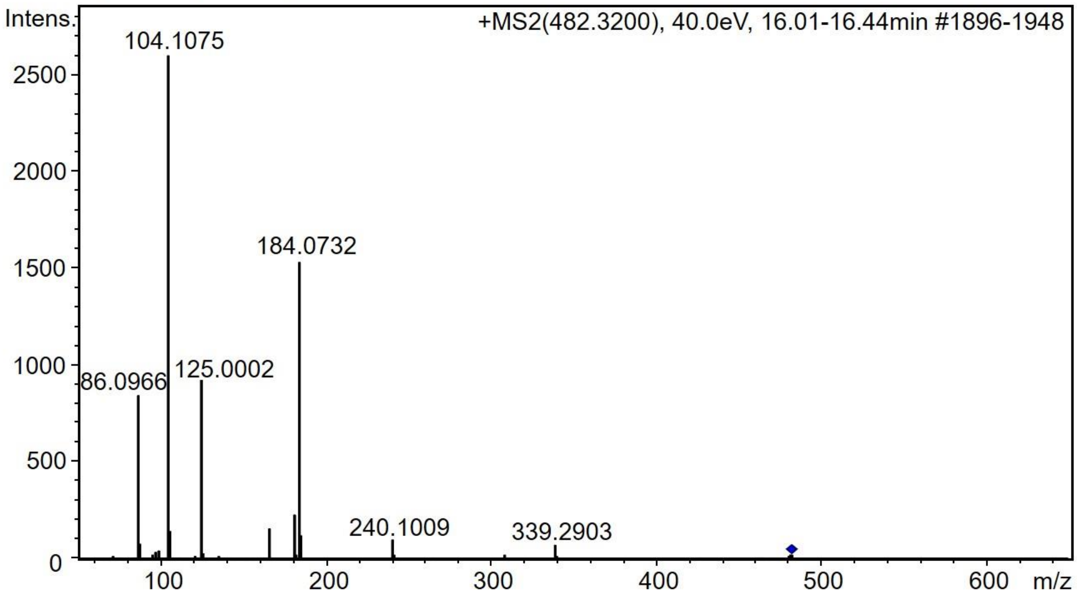


B LysoPC(16:0)

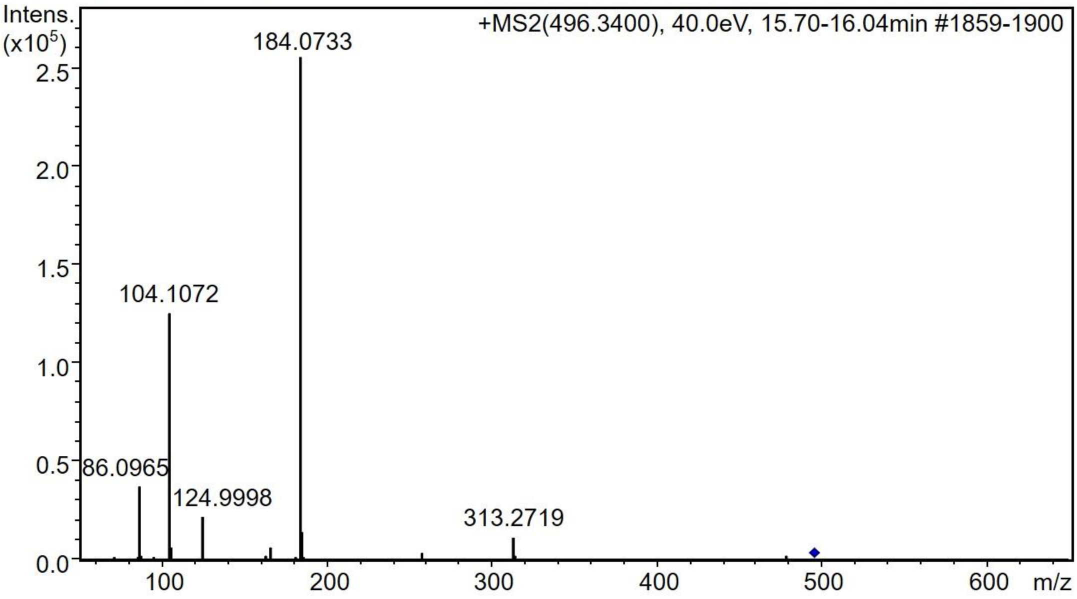

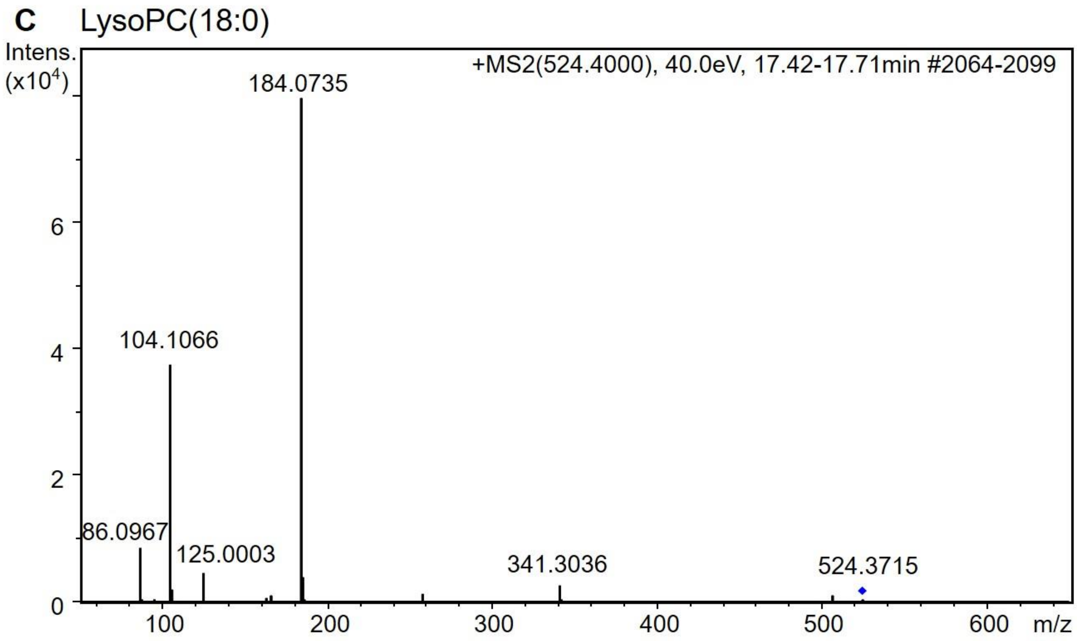
C
LysoPC(18:0)
Intens.
(x10^4)
+MS2(524.4000), 40.0eV, 17.42-17.71min #2064-2099
184.0735
104.1066
86.0967
125.0003
341.3036
524.3715
0
2
4
6
100
200
300
400
500
600
m/z

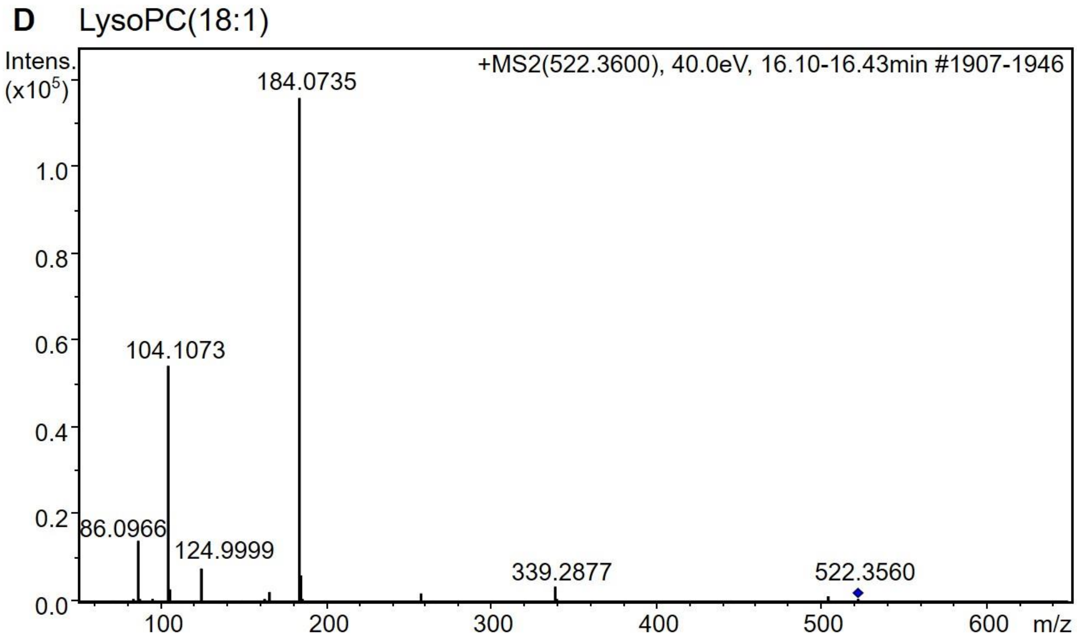
D
LysoPC(18:1)
Intens.
(x10^5)
+MS2(522.3600), 40.0eV, 16.10-16.43min #1907-1946
184.0735
104.1073
86.0966
124.9999
339.2877
522.3560
0.0
0.2
0.4
0.6
0.8
1.0
100
200
300
400
500
600
m/z

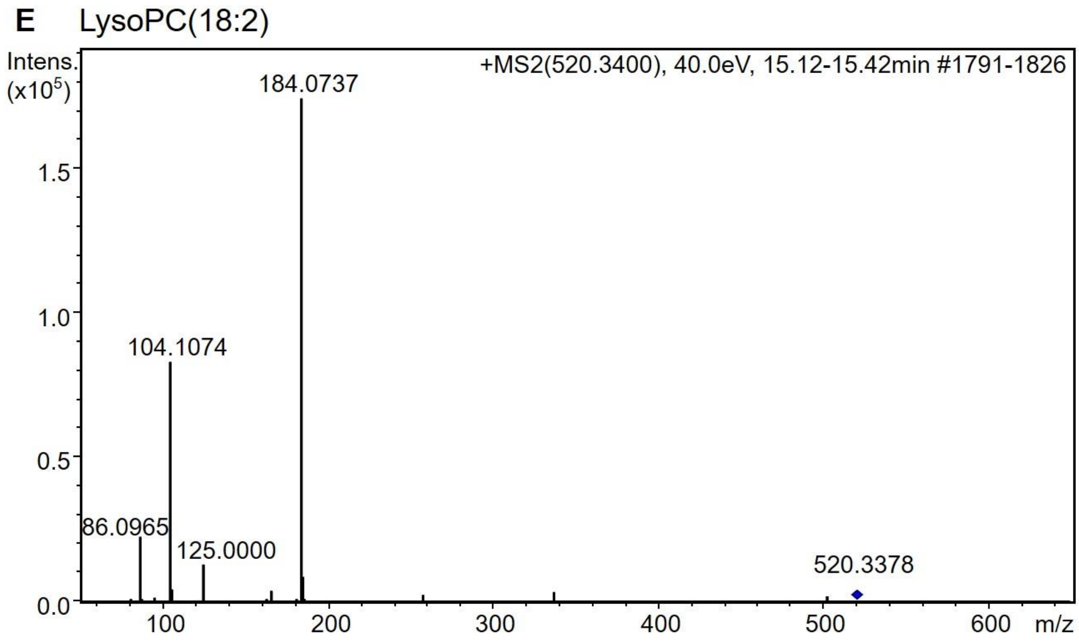


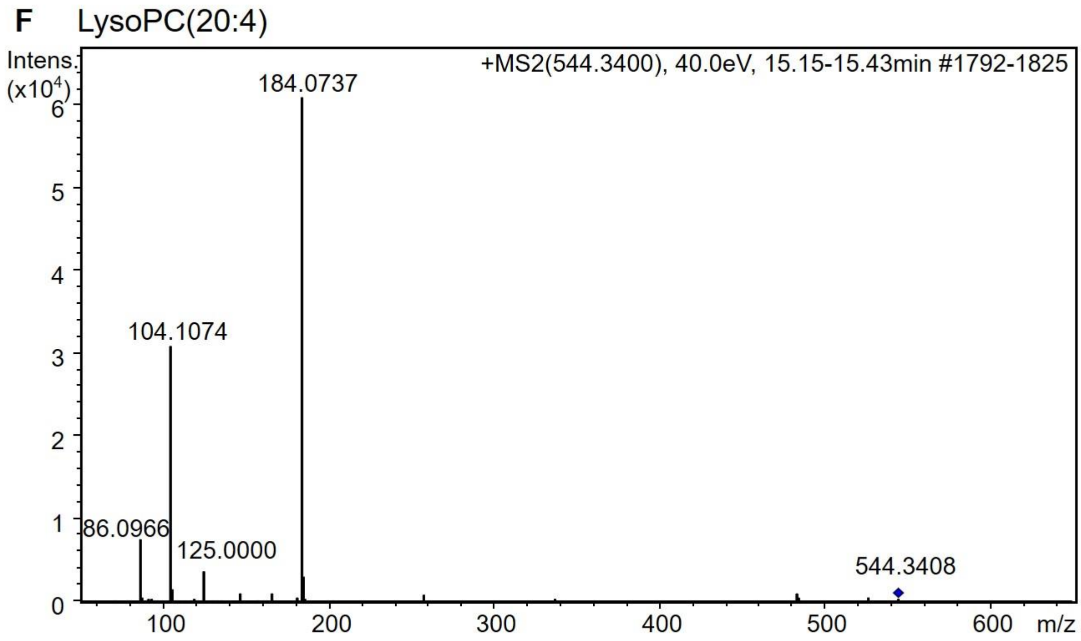


**S3 Fig. MS/MS spectra of lysophosphatidylcholines (lysoPCs).** Target ions were fragmented using nitrogen as collision gas and at a collision energy of 40 eV. (A) lysoPC(O-16:0), (B) lysoPC (16:0), (C) lysoPC (18:0), (D) lysoPC (18:1), (E) lysoPC (18:2), (F) lysoPC (20:4). Ester-linked and ether-linked lysoPCs provided characteristic fragment ions. Phosphocholine ($C_5H_{15}NO_4P^+$) ion, *m/z* 184.07; choline ($C_5H_{14}NO^+$) ion, *m/z* 104.11; *N*, *N*, *N*-trimethylethenaminium ($C_5H_{12}N^+$) ion, *m/z* 86.10; neutral loss of the phosphocholine group from ester-linked lysoPC $(M – C_5H_{15}NO_4P + H)^+$, *m/z* (protonated molecular ion – 183.07).
